# Use of Slag/Sugar Cane Bagasse Ash (SCBA) Blends in the Production of Alkali-Activated Materials

**DOI:** 10.3390/ma6083108

**Published:** 2013-07-25

**Authors:** Vinícius N. Castaldelli, Jorge L. Akasaki, José L.P. Melges, Mauro M. Tashima, Lourdes Soriano, María V. Borrachero, José Monzó, Jordi Payá

**Affiliations:** 1 UNESP–Univ Estadual Paulista, Campus de Ilha Solteira, Alameda Bahia 550, Ilha Solteira-SP CEP:15385-000, Brazil; E-Mails: vinicius_castaldelli@hotmail.com (V.N.C.); akasaki@dec.feis.unesp.br (J.L.A.); jlmelges@dec.feis.unesp.br (J.L.P.M.); 2 Instituto de Ciencia y Tecnología del Hormigón, Universitat Politècnica de València, Camino de Vera s/n, Edificio 4G, Valencia 46022, Spain; E-Mails: lousomar@upvnet.upv.es (L.S.); vborrachero@cst.upv.es (M.V.B.); jmmonzo@cst.upv.es (J.M.); jjpaya@cst.upv.es (J.P.)

**Keywords:** alkali-activation, sugar cane bagasse ash, slag replacement, waste valorization, microstructure, strength development

## Abstract

Blast furnace slag (BFS)/sugar cane bagasse ash (SCBA) blends were assessed for the production of alkali-activated pastes and mortars. SCBA was collected from a lagoon in which wastes from a sugar cane industry were poured. After previous dry and grinding processes, SCBA was chemically characterized: it had a large percentage of organic matter (*ca.* 25%). Solutions of sodium hydroxide and sodium silicate were used as activating reagents. Different BFS/SCBA mixtures were studied, replacing part of the BFS by SCBA from 0 to 40% by weight. The mechanical strength of mortar was measured, obtaining values about 60 MPa of compressive strength for BFS/SCBA systems after 270 days of curing at 20 °C. Also, microstructural properties were assessed by means of SEM, TGA, XRD, pH, electrical conductivity, FTIR spectroscopy and MIP. Results showed a good stability of matrices developed by means of alkali-activation. It was demonstrated that sugar cane bagasse ash is an interesting source for preparing alkali-activated binders.

## 1. Introduction

Concrete is certainly the most important construction material in the world. Its use is over 10 billion tons per year and, when done well, concrete can present good mechanical strength, and also, acceptable durability performance [1,2,3]. The main component of concrete is the binder that normally is composed of Portland cement, and in some cases, the presence of mineral additions, such as fly ashes or silica fume, can also be observed in its composition.

Portland cement is the conventional binding material that, actually, is responsible for about 5%–8% of global CO_2_ emissions. This environmental problem will most likely be increased due to exponential demand of Portland cement: By 2050, demand is expected to rise by 200% from 2010 levels, reaching 6000 million tons/year [4]. Out of concern for the environment, and in support of sustainable development, cement industries are improving their production through a range of alternatives such as, the use of alternative fuels or increasing the production of blended cements.

All these aspects have been contributing to reduce CO_2_ emissions, which can reach up to 30% of diminishing according to the Danish Centre for Green Concrete [5]. In this context, during the Copenhagen Summit held in 2009, different countries agreed on the necessity of reducing CO_2_ emissions by 2020. The United States, for example, made a pact to reduce its overall emissions by about 17% from 2010 in respect to the levels of 2005.

Hence, several research groups, and even the Portland cement industry, are investigating alternatives to produce green binding materials. Among these alternative materials, alkali-activated systems can be considered the most promising one due to its similar, or even better, mechanical properties and its high durability [6,7]. Moreover, these binding materials can reduce up to 80% of CO_2_ emissions when compared to that of Portland cement production [8,9].

Alkali-activated binders were for the first time investigated in 1957 [10], when Glukhovsky prepared a binder formed by mixing NaOH and slag. Actually, alkali activation is considered to be a polymerization reaction between an aluminosilicate source material and an alkaline solution to form a stable structure, and is always designated as an amorphous zeolite structure.

Aluminosilicate source materials commonly used for this purpose are blast furnace slag [11,12], fly ash [13,14] and metakaolin [15]. Nevertheless, other alumino-silicate materials can also be successfully employed in alkali-activated systems: glass fiber waste [16], ceramic waste [17,18], tungsten mine waste [19], hydrated-carbonated cement [20], fluid catalytic cracking catalyst residue (spent FCC) [21], air pollution control (APC) residues [22]. 

In some cases, the use of binary systems has been used in order to enhance the properties of alkali-activated systems formed [23,24]. Several studies related to alkali-activated systems based on slag/fly ash blends [23,25] and slag/metakaolin blends [26,27] are reported in the literature. 

Puertas *et al.* [28] reported a study of alkali-activated slag/fly ash cement, assessing different parameters that can influence the mechanical properties and the hydration products formed such as: alkaline concentration (2 and 10 M of NaOH), curing temperature (25 and 65 °C) and slag/fly ash ratio (100/0, 70/30, 50/50, 30/70, 0/100). Authors concluded that, depending on the parameters, compressive strength of about 50 MPa can be achieved, and related to the structure formed, the main reaction product is a CSH gel, with high amounts of thetracoordinated Al in its structure.

On the other hand, Bernal *et al.* [27] assessed the engineering properties of alkali-activated slag/metakaolin blends. Authors concluded that inclusion of metakaolin enhanced the compressive strength at early ages and this behaviour is favoured at high alkali concentrations.

Nowadays, several studies have been performed in order to reuse industrial and/or agricultural wastes abundantly generated in society: this approach is in agreement with sustainable development principles. Among the waste materials generated in Brazil, sugar cane bagasse is the most important in volume. The sugar cane production in Brazil is higher than 500 MTon per year, and part of the bagasse produced in the extraction of sugar and/or ethanol is usually exploited in furnaces for obtaining heat and water vapor. Nevertheless, this activity produces a final waste of 3 megatons of sugar cane bagasse ashes (SCBA).

There are many studies related to the reuse of SCBA as supplementary cementitious materials (SCM) in concrete and mortars [29,30,31]. The obtained results are very promising, but the amounts of SCBA used for this purpose constitutes 10%–20% of binder mass. The use of SCBA in alkali-activated systems was reported by Tippayasan *et al.* [32]. They found that 100% BA was inappropriate to produce geopolymers because of their low compressive strength. Some fly ash/SCBA mixtures were activated by means of 40% activating solution, and compressive strength values were in the 3–17 MPa range (cured at room temperature over 8 days). This behavior supported feasibility for the use of this type of mixture. Recently, Castaldelli *et al.* [33] reported a preliminary study using sugar cane bagasse ash in the production of alkali-activated binders, obtaining promising results. Hence, this paper assesses the mechanical and microstructural properties of alkali-activated binders based on slag/SCBA blends in different proportions: 100/0; 85/15; 75/25; 60/40.

## 2. Experimental Section

### 2.1. Materials

Blast furnace slag (BFS) was supplied by Cementval SL (Sagunto-Valencia, Spain). This hydraulic material was ground in a laboratory ball mill (alumina balls) for 30 min before its use. The mean particle diameter obtained for BFS was 21.4 μm. Sugar cane bagasse ash (SCBA) was collected from a settling lagoon in Destilaria Generalco S/A., close to General Salgado city (São Paulo, Brazil). The SCBA used in this study was obtained as follows:

i. uncontrolled burning of sugarcane bagasse to obtain heat;
ii. collection of ash generated by a scrubber;
iii. obtained ashes were mixed with water generated from sugar cane washing and then, deposited in the lagoon;
iv. settled solids from lagoon were collected and then dried at 105 °C;
v. collected ashes were ground in a laboratory ball mill (steel balls) for 20 min, obtaining a mean particle diameter of about 26.8 μm.

Some chemical reagents were used as alkaline activators: sodium hydroxide (98% purity, supplied by Panreac SA); and sodium silicate solution (supplied by Merck) with density of 1.35 g/cm^3^ and pH 11–11.5: its chemical composition (by mass) was: 8% Na_2_O, 28% SiO_2_ and 64% H_2_O.

### 2.2. Physico-Chemical and Mechanical Tests

Thermogravimetric Analysis (TGA) was performed in a TGA 850 Mettler-Toledo thermobalance. Pastes were analyzed under nitrogen atmosphere, using pin-holed aluminium sealed crucibles, with a heating rate of 10 °C min^−1^, from 35 °C until 600 °C. X-ray diffraction (XRD) studies were carried out in a Philips PW1710 diffractometer, using Cu-Kα wavelength, and 40 kV and 20 mA, in the 2θ range 5°–70°. Fourier transform infrared spectroscopy (FTIR) studies were performed in spectrometer Mattson Genesis II FTIR, which was connected to a computer, where the results were generated by the software WinFIRST FTIR. For this analysis, pellets of alkali-activated binder and KBr (1:200 sample/KBr mass ratio) were prepared. Samples for TGA, XRD and FTIR studies were prepared by grinding the paste with acetone, filtered, washed with acetone and dried at 60 °C in a furnace for 30 min.

A pHmeter Crison micropH2001 and a Crison microCM2201 conductimeter were used for measuring alkalinity of pastes [21]: 1 g of paste was ground and 10 mL of deionized water was added. After 10 min of continuous magnetic stirring, the pH and electrical conductivity were measured. Scanning electron microscopy (SEM) studies were carried out in a JEOL JSM-6300: samples were covered with gold. The mercury intrusion porosimetry (MIP) was performed on porosimeter AutoPore IV 9500 of Micrometrics Instrument Corporation with a range of pressures between 13,782 Pa and 227.4 MPa. Mortar and paste samples were evaluated at a pressure up to 0.21 MPa in the low pressure port, and 227.4 MPa in the high pressure port. Preparation of pastes and mortars: pastes were prepared mixing the binder and the corresponding activating solution. Mortars were prepared by addition of natural sand using a binder/sand ratio of 1/3. Mechanical strength tests were performed by using a universal test machine following the procedures described on UNE-EN 196-1. The flexural strength R_f_ value was the average of 3 specimens. The compressive strength R_c_ value was the average of 5 specimens (the sixth specimen was used for PIM analysis).

### 2.3. Preliminary Study Using BFS

The hydraulicity for BFS is well known, as well as the feasibility of its activation by addition of alkaline activators: sodium or potassium hydroxide [11,12] and waterglass [12,34]. A preliminary study was performed in order to show the importance of the nature of the alkali reagents. BFS was the mineral admixture used, and the following activations were carried out: pure water (solution 1), 5 mol kg^−1^ of sodium hydroxide (solution 2) and a mix of sodium hydroxide and sodium silicate, with 5 mol kg^−1^ of sodium cation and a SiO_2_/Na_2_O molar ratio of 1.46 (solution 3). In all cases, the water/BFS ratio was w/s = 0.45. Compressive strength on mortars (R_c_), and TGA and pH/conductivity on pastes were analyzed at 3 and 7 days of curing at 65 °C.

### 2.4. Study on Binders Containing SCBA

Mixtures containing BFS and SCBA were prepared by mixing (by mass):

- 100% BFS + 0% SCBA (mixture 100/0);
- 85% BFS + 15% SCBA (mixture 85/15);
- 75% BFS + 25% SCBA (mixture 75/25);
- 60% BFS + 40% SCBA (mixture 60/40).

The activating solution was prepared using sodium hydroxide and sodium silicate solution, and it had 5 mol^.^kg^−1^ of sodium cation, and presented a SiO_2_/Na_2_O molar ratio of 1.46 (this activating solution was selected from preliminary studies described above). The binder (BFS + SCBA) was mixed with the activating solution. Final water/(BFS + SCBA) ratio was 0.45. Two curing temperatures were used: 65 °C and 20 °C.

Pastes were stored in sealed plastic bottles at 65 °C for 3 and 7 days, and at 20 °C for 7, 28, 90 and 270 days. Mortars were cast in 160 mm × 40 mm × 40 mm prismatic molds (according to UNE-EN-196-1 standard). For highest curing temperature, molds were stored for 4 h at 65 °C in a water vapor saturated plastic box. Then, specimens were demolded and stored at 65 °C in the plastic box until mechanical testing. For the lowest curing temperature, molds were stored in a moist chamber (100% RH), and demolded after 24 h. The specimens were wrapped in plastic wrap and cured at 20 °C until mechanical testing. Prismatic specimens were tested after 3 and 7 days of curing at 65 °C, and after 7, 28, 90 and 270 days of curing at 20 °C.

## 3. Results and Discussion

### 3.1. Chemical and Mineralogical Characterization of BFS and SCBA

The chemical compositions of blast furnace slag (BFS) and sugar cane bagasse ash (SCBA) are shown in Table 1.

**Table 1:** Chemical composition of Blast Furnace slag (BFS) and Sugar Cane Bagasse Ash (SCBA).

Oxide	BFS	SCBA
SiO_2_	30.19	31.41
Al_2_O_3_	10.66	7.57
Fe_2_O_3_	1.31	6.02
CaO	39.53	16.06
MgO	7.50	1.07
Na_2_O	0.87	0.14
K_2_O	0.58	1.58
SO_3_	1.95	0.78
TiO_2_	0.51	2.09
MnO	0.40	0.10
Chloride	0.44	0.14
LOI	5.62	32.20

**Figure 1:**
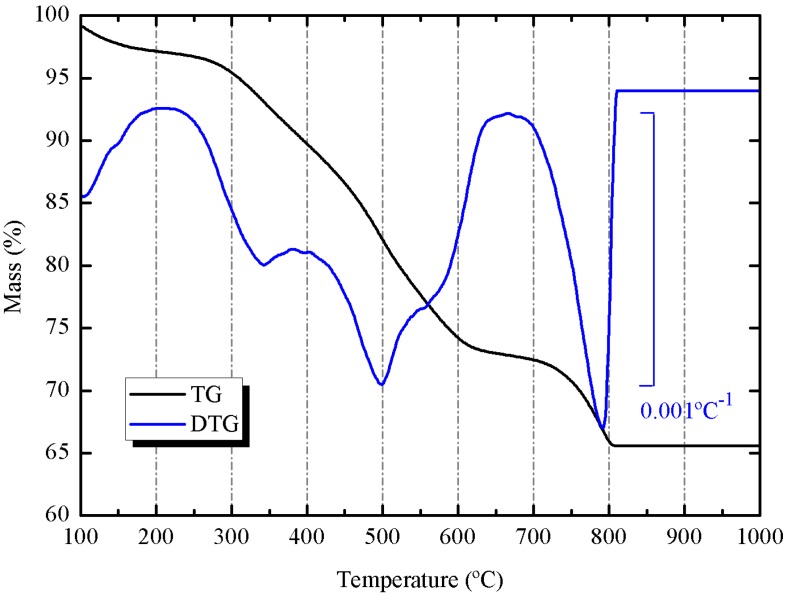
Thermogravimetric (TG) and derivative thermogravimetric (DTG) curves for SCBA: heating rate 20 °C/min, 70 μL alumina crucible, dried air atmosphere.

SCBA presented a high percentage loss on ignition (32.2%). This fact is attributed to mixing the liquid from gas scrubber and the wastewater from washing sugarcane, which contains a high amount of organic matter. The Figure 1 shows the thermogravimetric curve for the ash (heated in air at 20 °C/min heating rate, using alumina crucible). It is important to notice that part of the mass loss (24.68%) was produced in the 250–650 °C range, which belongs to organic matter volatilization and oxidation. However, a part of the mass loss (6.86%) was observed at 700–800 °C range, which corresponds to the decomposition of calcium carbonate. The percentage of CaCO_3_ calculated from this mass loss was 15.59%.

**Figure 2:**
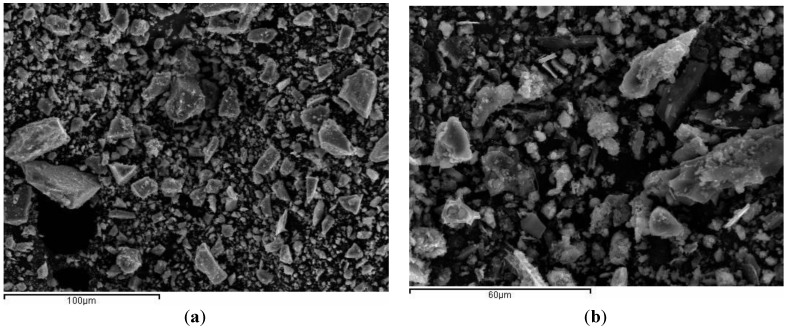
SEM micrographs: (**a**) BFS and (**b**) SCBA.

The particle morphology for BFS is depicted in Figure 2a. Particles present fairly dense, smooth texture, sharp particles and different sizes. Particle morphology of ground SCBA is shown in Figure 2b. It can be seen that particles are irregular in shape. Spherical shaped particles were not found, suggesting that the combustion temperature reached in the burning process did not produce the melting of inorganic matter. SCBA particles presented rough surfaces.

**Figure 3:**
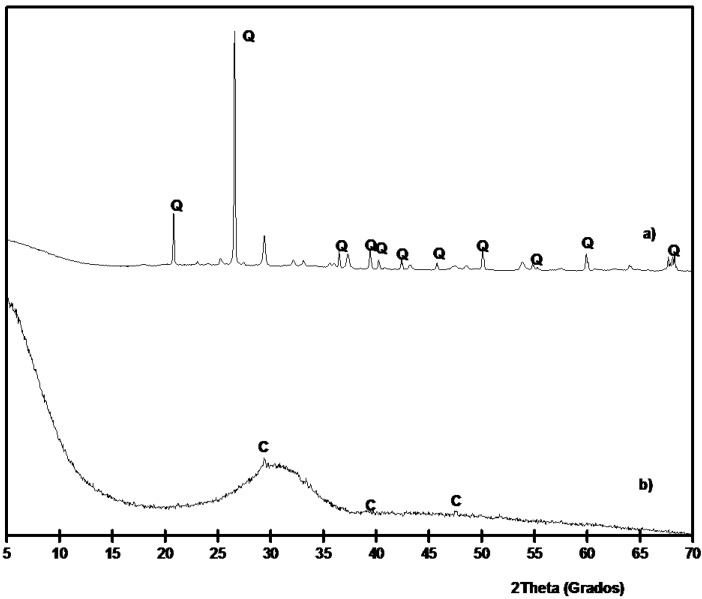
XRD diffractograms for: (**a**) SCBA; (**b**) BFS. (Key: Q: Quartz; C: Calcite).

SCBA and BFS were characterized by means of XRD analysis. The corresponding diffractograms are shown in Figure 3. It is noticeable the high crystallinity degree of SCBA: the baseline of the diffractogram had not deviated in the 2θ range 20°–40°, suggesting that the proportion of crystallized fractions is important. The insoluble residue was determined for SCBA by means of dissolution in refluxing 4 M potassium hydroxide [35]. The obtained value was 24.1% ± 0.6%: the residue was due to the presence (see Figure 3) of quartz (PDF card 331161) as the main crystallized compound; also calcite was identified (PDF card 050586). The background level for BFS is higher than those found for SCBA. Additionally, BFS had a very important vitreous fraction, accordingly to the baseline deviation in the 20°–35° 2θ range. A trace of calcite was identified in its XRD spectrum.

**Figure 4:**
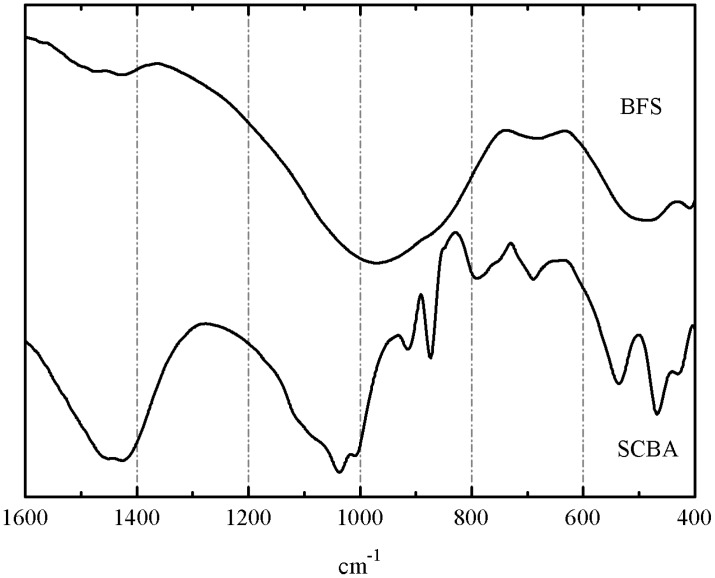
FTIR spectra for SCBA and BFS (KBr pellets).

FTIR spectra for BFS and SCBA are depicted in Figure 4. The spectrum for BFS showed a broadband characteristic of gehlenite. Two strong peaks are noticed: one of them centered at 981 cm^−1^, attributed to symmetric stretching vibration of Si(Al)–O–Si bonds, and another one at 527 cm^−1^, belonging to in-plane bending Si(Al)–O–Si vibrations of aluminosilicate network [36]. Small peaks attributed to carbonate anion vibrations (*ca.* 1430 and 710 cm^−1^) were also identified. The spectrum of SCBA showed more peaks: the highest intensity absorption peak was related to the Si(Al)–O–Si network: a intense a broad band centered at 1030–1050 cm^−1^ (asymmetric stretching vibration of Si(Al)–O–Si bonds). Also, peaks corresponding to quartz were noticed at 792 and 468 cm^−1^. Additionally, peaks belonging to carbonate anion (from calcite) were also identified: 1437 cm^−1^ (asymmetric stretching vibration of CO_3_^2−^ anion) and 873 cm^−1^ (out-of-plane bending mode of CO_3_^2−^) [36]. Sharp peaks at 1035, 914, 663, and 538 cm^−1^ were attributed to organic matter presence in the ash, probably due to C–O stretching in alcohol groups or other oxygen-containing functional groups, out of plane C=C–H bending, out of plane C≡C–H bending, and out of plane aromatic ring bending vibrations [37]. These peaks are attributed to organic compounds disappearing in FTIR after calcination of SCBA at 650 °C.

### 3.2. Preliminary Results

**Figure 5:**
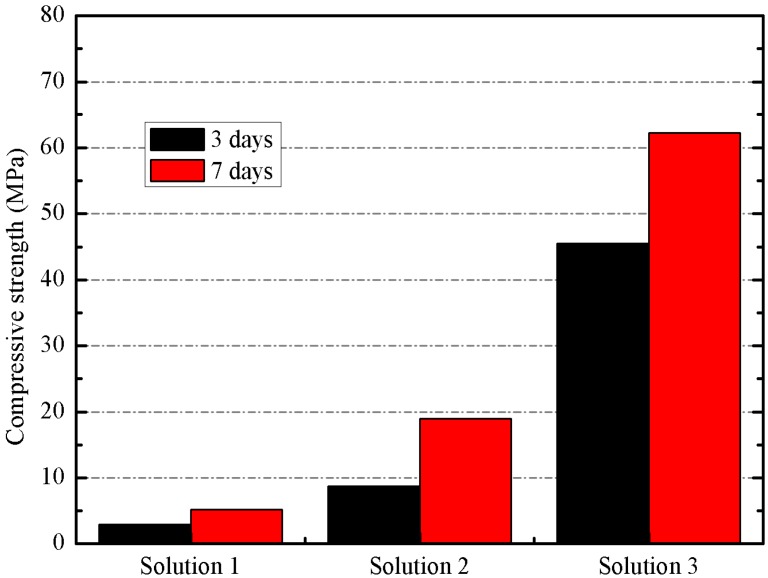
Compressive strength of mortars activated with different activating solutions.

Three mortars (100% BFS as mineral admixture) were prepared by using different solutions: pure water (solution 1); 5 mol kg^−1^ of sodium cation (solution 2); and 5 mol kg^−1^ of sodium cation and a SiO_2_/Na_2_O molar ratio of 1.46 (solution 3). They were cured at 65 °C and tested in compression after 3 and 7 days. In Figure 5, the compressive strength values of mortars activated with different activating solutions is shown.

On one hand, the significant increase in compressive strength values at both ages justifies the alkaline activation of BFS by using a mixture of solid NaOH and sodium silicate solution.

**Figure 6:**
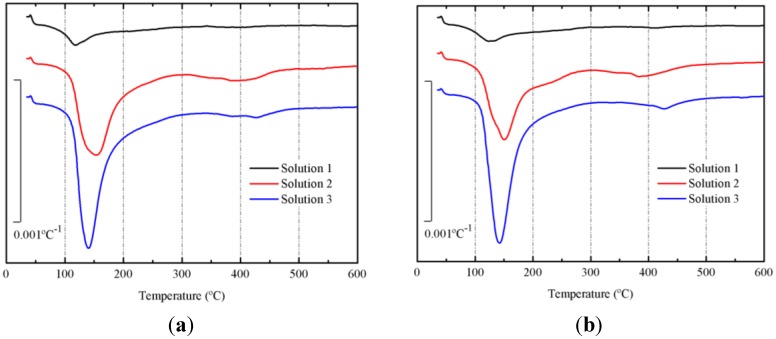
Derivative thermogravimetric curves (DTG) for preliminary study on BFS pastes, after curing at 65 °C: (**a**) 3 days; (**b**) 7 days.

Derivative thermogravimetric curves (DTG) of pastes cured at 65 °C for 3 and 7 days are depicted in Figure 6a,b, respectively. In both figures (for all curves), a main peak centered at the 135–145 °C range is noticed. This peak belongs to the dehydration/dehydroxylation process [21] for the gels formed in the alkali activation of BFS. The following mass losses after 3 days were calculated: 4.51% for paste with solution 1, 16.24% for paste with solution 2 and 18.15% for paste with solution 3. And after 7 days, the mass losses were respectively: 5.13%, 16.62% and 19.28%. Pastes prepared with solution 3 had the highest mass loss, suggesting that using this solution, a more important progression in the alkali activation of BFS is shown. Additionally, the increase in the mass loss with curing time, indicated that the reaction took place also in the 3–7 day period.

Paste produced with solution 1 presented the lowest alkalinity, pH = 11.91 after 3 days of curing and pH = 11.92 after 7 days of curing. Paste prepared with solution 2 presented pH = 12.96 after 3 days of curing and pH = 12.90 after 7 days of curing, and the paste with solution 3 had pH = 12.85 and pH = 12.80 respectively. The pH of the paste with solution 1 is lower than others, because it was activated by plain water: the alkalinity was due to hydraulicity of BFS. The pH values for BFS pastes activated with solutions 2 and 3 were significantly lower than initial pH of the corresponding solutions, suggesting that an important amount of hydroxyl anions was chemically combined with mineral compounds in BFS, and this means dissolution and precipitation of the gel [21].

For the following section, solution 3 was selected for activating mineral admixtures containing BFS and SCBA. The main reasons for this selection were the higher amount of chemically combined H_2_O/OH^−^ groups in the formed gel and the development in the compressive strength of mortars.

### 3.3. Results on Binders Containing SCBA

All mixtures were prepared and activated with a solution of 5 mol kg^−1^ of sodium cation, a SiO_2_/Na_2_O molar ratio of 1.46 and a water/binder ratio of 0.45. Pastes cured for 3 days at 65 °C were characterized by means of SEM, TGA, XRD, pH and FTIR. TGA curves were also analyzed on the pastes cured for 7 days at 65 °C. Pastes for 28–270 days of curing at 20 °C were characterized by TGA, XRD, pH and FTIR. Mortars were mechanically characterized (compressive and flexural strengths) at 3 and 7 days of curing at 65 °C and at 7, 28, 90 and 270 days of curing at 20 °C. MIP tests were carried out on mortars at 3 days of curing at 65 °C and 270 days of curing at 20 °C, and also on pastes cured for 270 days at 20 °C.

**Figure 7:**
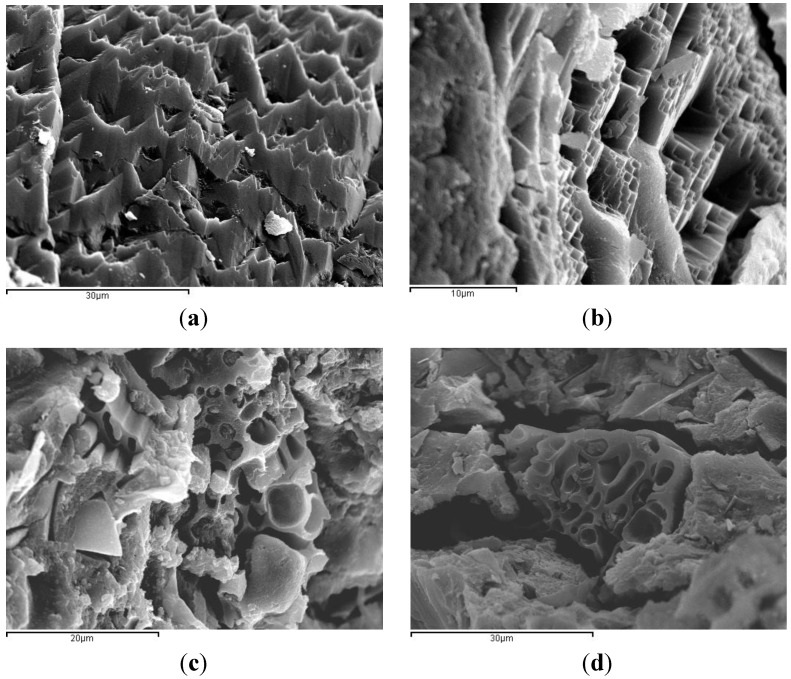
SEM micrographs of alkali-activated binders of BFS + SCBA cured at 65 °C for 3 days: (**a**) mix 100/0; (**b**) mix 85/15; (**c**) mix 75/25; (**d**) mix 60/40.

SEM micrographs of BFS/SCBA pastes cured at 65 °C for 3 days are shown in Figure 7. The Figure 7a shows the mix 100/0: a dense structure with sharp shapes and with some small pores. The Figure 7b shows the mix 85/75: a similar gel structure was observed, as above. The dense matrix found in mixes 100/0 and 85/15 may be a consequence of the activation of the mineral admixture. Figure 7c shows the mix 75/25: it was noticed a less dense structure quite different from the above pastes. Some porous particles embedded in the gel matrix were observed, due to the presence of unreacted SCBA particles (unburned or partially unburned bagasse particles). And finally, the Figure 7d shows the mix 60/40, very similar to mix 75/25. Apparently, the highest contents of SCBA (25% and 40%) produced a more porous matrix.

**Figure 8:**
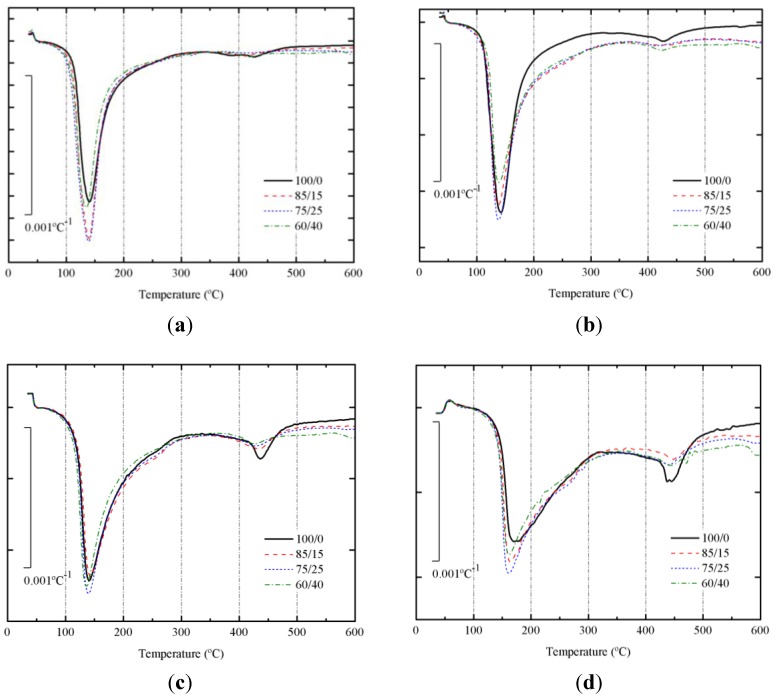
DTG curves for alkali activated BFS + SCBA pastes cured: (**a**) after 3 days at 65 °C; (**b**) after 7 days at 65 °C; (**c**) after 28 days at 25 °C; (**d**) after 270 days at 25 °C.

DTG curves for SCBA + BFS pastes cured at 65 °C for 3 days and 7 days are depicted in Figure 8a,b, respectively. Corresponding DTG curves for pastes cured at 20 °C for 28 and 270 days are depicted in Figure 8c,d, respectively. Table 2 summarizes the total mass loss for all these pastes in the 35–600 °C range.

**Table 2:** Total mass losses in the 35–600 °C range for BFS + SCBA pastes and temperature at the highest mass loss rate.

Mix BFS/SCBA	Mass loss in pastes in different curing conditions (days–temperature) and temperature at the highest mass loss rate (°C, in parentheses)			
	3 d–65 °C	7 d–65 °C	28 d–20 °C	270 d–20 °C
100/0	18.15 (140)	19.28 (143)	15.69 (141)	15.58 (171)
85/15	20.00 (139)	17.93 (139)	16.36 (143)	15.87 (164)
75/25	21.42 (139)	18.34 (138)	16.77 (140)	17.25 (162)
60/40	19.15 (135)	17.53 (139)	16.33 (136)	16.81 (161)

In all DTG curves, a peak centered in the 135–171 °C range was observed. This behavior means that the alkaline activation took place and some binder gel was formed [16,33]. The water molecules and OH groups are bonded to the new aluminosilicate network. Mass losses for pastes cured at 65 °C were higher than those found for pastes cured at 20 °C. This fact means that the matrix formed at 65 °C presented more H_2_O/OH groups. And the DTG peak did not shift after increasing the curing time from 3 to 7 days. Also, mass losses for pastes cured at 20 °C did not vary from 28 to 270 days; however, in this case a significant shift of the DTG peak was observed, from the 136–143 °C to the 161–171 °C range. This behavior would be related to the stronger binding of H_2_O/OH groups in the matured matrix for long curing times. These data suggest that it would be an interesting change in mechanical properties of these matrices.

Additionally, a small peak was identified in the 420–470 °C range. This peak is more important for pates cured at 65 °C for 7 days, and especially for pastes cured at 20 °C for 270 days. Moreover, this peak is larger for pastes 100/0, suggesting that this peak could be related to the presence of slag in the studied pastes. Probably, the decomposition observed at this temperature range is related to the presence of brucite or hydrotalcite [34,38,39].

**Figure 9:**
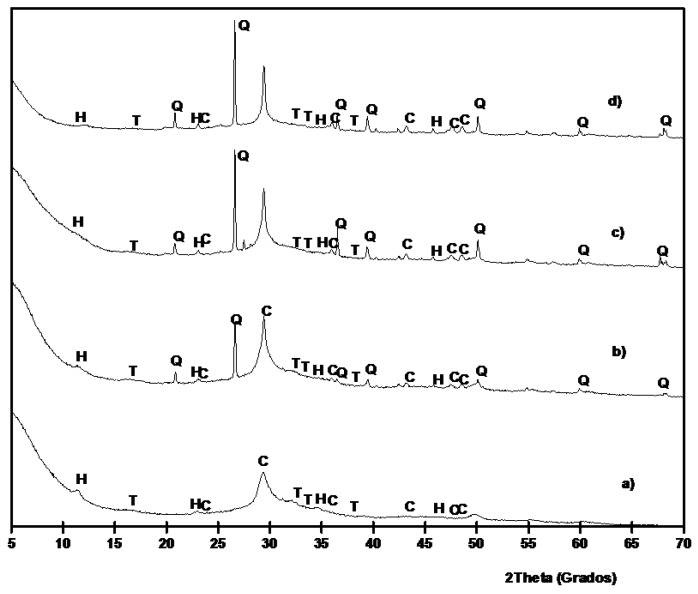
XRD diffractograms for BFS/SCBA pastes cured for 3 days at 65 °C: (**a**) 100/0; (**b**) 85/15; (**c**) 75/15; (**d**) 60/40. (Key: Q: Quartz; C: Calcite; T: Thermonatrite; H: Hydrotalcite).

XRD patterns for 100/0 paste cured for 3 days at 65 °C is shown in Figure 9a. The most important peak present in this paste is a broad peak centered at 2θ = 29.35, which is slightly lower than those found in BFS (centered at 2θ = 30.86°). This behavior demonstrates the formation of an amorphous gel C–N–S–A–H [13,40]. Also, peaks belonging to calcite, thermonatrite (Na_2_CO_3_·H_2_O, PDF card 080448) and hydrotalcite (PDF card 140191) were identified. The presence of hydrotalcite, Mg_6_Al_2_CO_3_(OH)_16_·4H_2_O, agree with TGA identification. For pastes containing SCBA (Figure 9b–d) the presence of quartz and calcite became more important, because of the replacement of BFS by SCBA. The baseline deviation for BFS/SCBA mixtures was less important because the presence of quartz and calcite. Also, traces of hydrotalcite were found.

**Figure 10:**
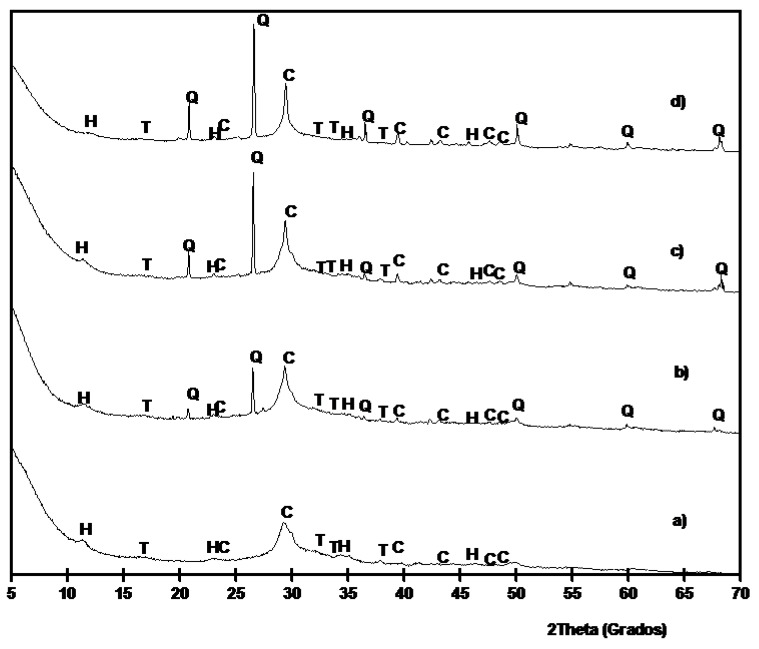
XRD diffractograms for BFS/SCBA pastes cured for 270 days at 20 °C: (**a**) 100/0; (**b**) 85/15; (**c**) 75/15; (**d**) 60/40. (Key: Q: Quartz; C: Calcite; T: Thermonatrite; H: Hydrotalcite).

Figure 10 shows the XRD diffractograms for pastes cured at 20 °C for 270 days. Similar results were obtained if compared to results on pastes cured at 65 °C. In this case, hydrotalcite peaks were easily observed.

**Figure 11:**
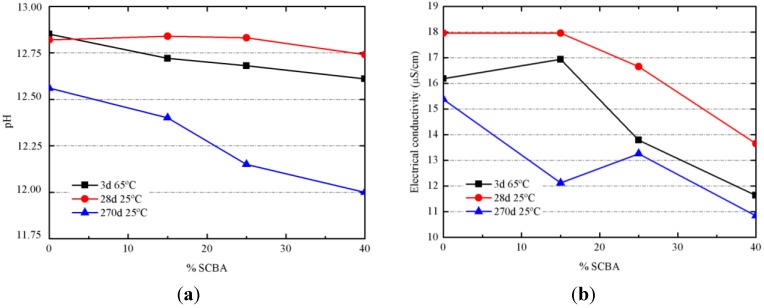
Evolution of the properties of alkali-activated pastes: (**a**) pH values; (**b**) electrical conductivity values.

The progress of alkali-activated reaction was monitored by means of pH and electrical conductivity measurements in an aqueous suspension [21]. Pastes cured after 3 days at 65 °C and pastes cured after 28 days at 20 °C had small differences on pH when the replacing percentage of SCBA was increasing (Figure 11a). For pastes cured at 20 °C for 270 days, differences are more significant, finding that 100/0 had pH = 12.56; pH values for 85/15, 75/25 and 60/40 were lowered to 12.40, 12.15 and 12.00, respectively. This behavior has been attributed to two factors: firstly, the hydraulicity of BFS, which favored the increase of pH; and secondly, the reactivity of SCBA by combination of silica network with OH^−^ anions, by the cleavage of Si–O–Si bonds to produce silanol groups (Si-O-H). The higher chemical reaction progress for pastes cured at 20 °C for 270 days will be assessed by means of mechanical experiments (see below). Associated to the pH reduction, there is a parallel decrease of electrical conductivity (Figure 11b). Alkali-activator solution was the same for all pastes, and consequently, the lowest electrical conductivity for pastes containing the highest replacement of BFS by SCBA suggests that a larger quantity of ions (sodium cation, silicate and hydroxyl anions) were chemically reacted.

**Figure 12:**
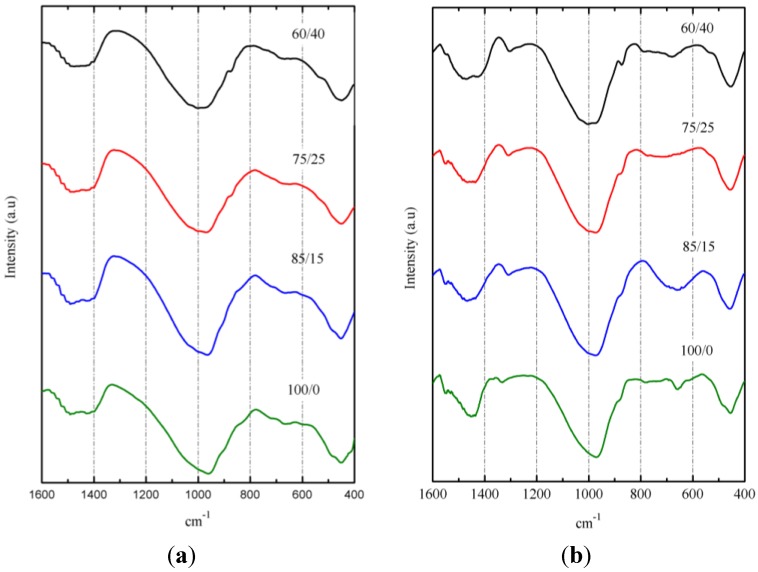
FTIR spectra for BFS/SCBA pastes: (**a**) cured at 65°C for 3 days; (**b**) cured at 20 °C for 270 days.

Figure 12 shows the FTIR spectra of pastes after 3 days of curing at 65 °C (Figure 12a) and after 270 days of curing at 20 °C (Figure 12b). The broadness of the main absorption band (Si–O stretching vibrations) around 960–973 cm^−1^ for pastes cured at 65 °C and 974–1004 cm^−1^ for pastes cured at 20 °C is indicative of the disordered structure of these materials. According to Clayden *et al.* [41], a broadness of the main band results from the coexistence of various SiQ^n^ units in the amorphous network. The peak of this broad band shifted to lower wave-number values for 100/0 pastes, probably due to the presence of more aluminum in BFS than in SCBA [42]. The increasing curing time from 3 to 270 days resulted in shifting the main band (e.g., for the 100/0 paste, the shift was from 960 to 974 cm^−1^, and for the 60/40 paste, the shift was from 974 to 1004 cm^−1^. This could be a consequence of increasing Q^3^ units [36] and can be attributed to the role of SCBA.

Table 3 shows mechanical strengths values (compressive, R_c_; flexural, R_f_) of mortars cured at 65 °C after 3 and 7 days of curing. R_c_ values at 3 days were in the 42–54 MPa range, finding higher R_c_ values for 85/15 and 75/25 mixtures. For 7 days curing time, 100/0 sample increased the R_c_ value to 62.2 MPa, whereas mortars containing SCBA showed little change.

This behavior could be attributed to the SCBA that contribute on the early stages of alkali activation reaction (in the first 3 days). However, the sample with only BFS as a mineral admixture, present a progress on the strength development from 3 to 7 days, as observed in TG analysis of pastes. In terms of R_f_ values, a decrease was observed from 3 to 7 days, suggesting changes in the microcrack pattern due to prolonged high temperatures, specially for SCBA containing mortars. In these conditions (high curing temperature), the negative influence of organic matter and carbon present in SCBA on the hardening process of alkali-activated systems based on Slag/SCBA is negligible.

**Table 3:** Mechanical strengths of mortars cured at 65 °C.

Mixtures	R_c _(MPa)		R_f _(MPa)	
	3 days	7 days	3 days	7 days
100/0	45.5 ± 2.9	62.2 ± 2.6	5.80 ± 0.3	5.39 ± 1.1
85/15	53.5 ± 2.0	51.2 ± 0.4	5.31 ± 0.4	2.94 ± 0.6
75/25	49.0 ± 2.7	52.8 ± 1.9	5.31 ± 0.6	4.00 ± 0.4
60/40	42.8 ± 0.9	43.2 ± 0.3	3.84 ± 0.5	3.19 ± 0.4

**Figure 13:**
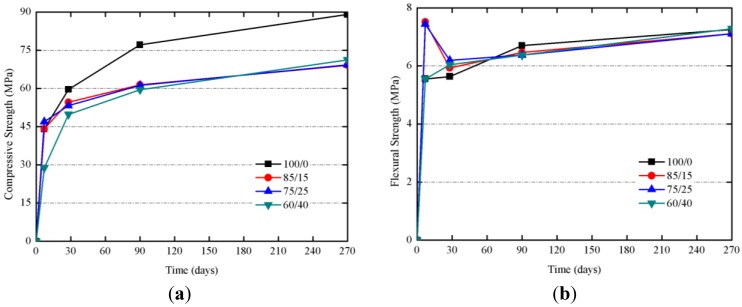
Mechanical strength developments for of BFS + SCBA mortars cured at 20 °C: (**a**) Compressive strength; (**b**) Flexural strength.

Studies on mortars cured at 20 °C at 7, 28, 90 and 270 days were carried out. The Figure 13a shows the evolution for R_c_ of samples cured at 20 °C. R_c_ values at 28 days were very similar, and for longer curing times (90 and 270 days), 100/0 mortar showed a significant increase on R_c_ (from 59.6 to 89.0 MPa), suggesting that, on one hand, the presence of SCBA enhances the reactivity at early ages, and, on the other hand, the matrix containing only BFS is developed for longer ages. Samples containing SCBA showed very similar R_c_ values after 270 days (*ca.* 70 MPa), values significantly higher than those found for mortars cured at 65 °C (43–53 MPa, see Table 2) indicating that the curing at lower temperatures let to form better developed matrices. Once again, it has been observed that the presence of organic matter and carbon in SCBA did not adversely influence strength development, even at lower curing temperatures. Also, interestingly, R_f_ values (see Figure 13b) were in the 6–8 MPa range for longest the curing times: in this case, no decay in R_f_ values was observed, suggesting that the matrix produced at 20 °C did not suffer critical microcracks.

Finally, cured samples were characterized by means of Mercury Intrusion Porosimetry (MIP). The test was conducted for mortars cured for 3 days at 65 °C. In Table 4, selected data for all mortars are summarized. Sample 100/0 showed the smallest total porosity, this indicates that the presence of the SCBA in the alkali activated binders did not contribute to reduce the total volume of accessible pores in this type of test. In general terms, for all pore size range, mortars containing SCBA showed higher specific volume of Hg (mL of Hg/g of mortar). The Hg retained after the extrusion step was high in all samples, suggesting that the alkali-activated matrices presented a significant tortuosity degree.

In Table 5, data for mortars cured at 20 °C over 270 days are summarized. In this case, the total porosity obtained was lower than that found for mortars cured at 65 °C. This fact suggests that, taking into account that dosage compositions were the same in both curing conditions, the reduction in curing temperature allowed an improved development of the matrix, closing many pores and capillaries, and then reducing the total volume of pores. This reduction was found for all selected pore size ranges summarized in Table 5. Also, for these mortars, the mercury retained in the extrusion process was high: in this curing condition, the percentage of Hg retained was higher for samples with a large amount of SCBA (75/25 and 60/40), suggesting the importance of the role of SCBA particles in the development of the matrix. This behavior was also found for pastes cured at 20 °C for 270 days (Table 6). In this case, the total porosity was higher for all tested samples if compared to those found for mortars. In general terms, the total volume of capillary pores (1 μm to 10 nm) was higher for samples containing SCBA particles, and also for the volume of gel pores.

**Table 4:** MIP results for mortars cured 3 days at 65 °C.

Mixtures	Total porosity (%)	Total pore area (m^2^/g)	Median pore diameter		Volume (mL of Hg/g of mortar)				Hg retained (%)
			Volume (nm)	Area (nm)	>1 μm	1 μm–50 nm	50–10 nm	<10 nm	
**100/0**	9.43	0.251	15,683.0	5.8	0.0381	0.0019	0.0001	0.0004	81.64
**85/15**	12.58	1.918	17,706.1	7.2	0.0517	0.0033	0.0006	0.0032	86.53
**75/25**	9.82	2.897	7154.3	6.9	0.0351	0.0047	0.0008	0.0047	74.35
**60/40**	11.30	5.321	4823.1	6.6	0.0375	0.0070	0.0018	0.0083	70.59

**Table 5:** MIP results for mortars cured after 270 days at 20 °C.

Mixtures	Total porosity (%)	Total pore area (m^2^/g)	Median pore diameter		Volume (mL of Hg/g of mortar)				Hg retained (%)
			Volume (nm)	Area (nm)	>1 μm	1 μm–50 nm	50–10 nm	<10 nm	
**100/0**	6.80	2.070	10,813.8	8.2	0.0229	0.0024	0.0021	0.0021	71.29
**85/15**	7.48	1.154	8835.9	8.2	0.0278	0.0037	0.0008	0.0017	77.57
**75/25**	7.62	1.989	6903.3	7.3	0.0256	0.0051	0.0010	0.0032	75.64
**60/40**	9.61	1.535	8554.4	7.6	0.0348	0.0064	0.0011	0.0021	84.35

**Table 6:** MIP results for pastes cured after 270 days at 20 °C.

Mixtures	Total porosity (%)	Total pore area (m^2^/g)	Median pore diameter		Volume (mL of Hg/g of mortar)				Hg retained (%)
			Volume (nm)	Area (nm)	>1 μm	1 μm–50 nm	50–10 nm	<10 nm	
**100/0**	8.78	1.287	7738.9	6.4	0.0405	0.0033	0.0001	0.0021	69.53
**85/15**	9.69	5.713	1423.5	7.4	0.0319	0.0127	0.0032	0.0092	74.13
**75/25**	8.60	3.173	1933.6	6.7	0.0339	0.0109	0.0043	0.0048	83.97
**60/40**	12.53	3.881	1125.6	7.7	0.0427	0.0306	0.0030	0.0053	83.15

## 4. Conclusions

The sugar cane bagasse ash (SCBA) studied presented a high percentage of crystallized material (mainly quartz, also calcite) and a high proportion of organic matter (*ca.* 25%). Despite this, the amount of soluble material in alkaline conditions suggested that it could be an interesting waste material for producing alkali-activated binders. Alkali-activated binders based on slag/SCBA blends were prepared and their microstructure, their physico-chemical properties and their mechanical strength development were assessed. Sodium hydroxide and a waterglass mixture were selected for activating BFS/SCBA samples: 5 mol kg^−1^ of sodium cation and a SiO_2_/Na_2_O molar ratio of 1.46. Mineral BFS/SCBA mixtures were dosed in the following proportions by weight: 100/0, 85/15, 75/25 and 60/40. Studies on pastes and mortars cured for 3–7 days at 65 °C demonstrated that there was an important reaction degree of SCBA particles in the formation of gel matrices, and a good contribution on compressive strength was measured: SCBA containing mortars with 42–54 MPa after 3 days of curing at 65 °C were obtained. The development of BFS/SCBA blends alkali-activated matrices cured at 20 °C was better than at 65 °C: the H_2_O/OH groups in the gel formed were strongly bonded according to the thermogravimetric analysis. Moreover, mortars yielded high strengths after long curing times (90 and 270 days): compressive strengths in the 55–65 MPa range were obtained. In the same way, the porous structure of mortars was enhanced for mixtures cured at 20 °C, yielding a reduction in total porosity to 7.5%–10%, clearly lower than those found for mortars cured at 65 °C (9.5%–12.5%). In general terms, this study demonstrates the feasibility of the use of slag/SCBA blends in alkali-activated systems, and these types of mixtures could form part of an alternative approach to reusing ashes obtained in the sugar cane industry.
